# Investigation of the AgCl Formation Mechanism on the Ag Wire Surface for the Fabrication of a Marine Low-Frequency-Electric-Field-Detection Ag/AgCl Sensor Electrode

**DOI:** 10.1021/acsomega.2c01481

**Published:** 2022-06-24

**Authors:** Kang Rae Cho, Minhye Kim, Bupmo Kim, Gahye Shin, Sangkyu Lee, Wooyul Kim

**Affiliations:** † Department of Energy Engineering/KENTECH Institute for Environmental and Climate Technology, Korea Institute of Energy Technology (KENTECH), Naju 58330, Republic of Korea; ‡ Department of Chemical Engineering & Division of Environmental Science and Engineering, Pohang University of Science and Technology, Pohang, Gyeongbuk 37673, Republic of Korea; § Maritime Technology Research Institute 1st Directorate Agency for Defense Development, Jinhae-gu, Changwon-si 51698, Republic of Korea

## Abstract

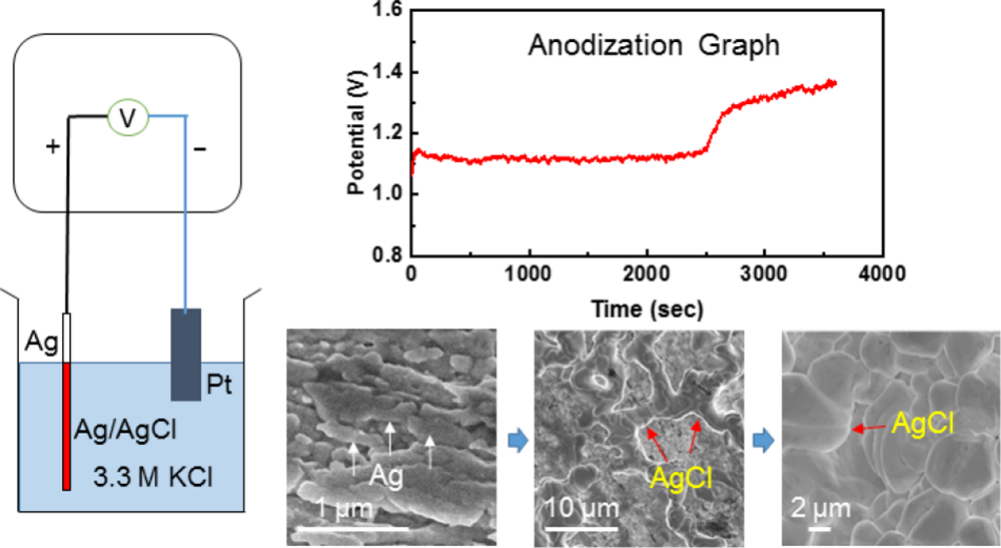

One of the most widely used electric field sensors for low-frequency electric field detection (LFEFD) in seawater uses the Ag/AgCl electrode. The surface structure of the electrode including AgCl layers plays a critical role in the electrode’s electrochemical performance required for the sensor. In this study, the sequential AgCl formation process under the constant current was examined on the Ag wire in an electrode size for actual applications, and an optimal electrode surface structure was suggested for the LFEFD Ag/AgCl sensor. Upon mild anodization (0.2 mA/cm^2^) in 3.3 M KCl solution that permits us to follow the AgCl formation process manageably, Ag dissolution from the wire surface begins leaving cavities on the surface, with the accompanied growth of initial Ag grains. During this period, AgCl deposits in sizes of about several micrometers to 10 μm with crystal planes also form primarily along scratch lines on the wire surface, but in a partial scale. Then, with further anodization, the assumed thin AgCl deposits start to form, covering a large portion of the wire surface. They grow to become deposits in sizes of about several micrometers to 10 μm with no clear facet planes next to one another and are connected to form the network structure, representing the main developing mode of the AgCl deposits. While they cover all the surface, AgCl deposits also form on the surface of the already formed ones, making multiple AgCl layers. All these deposits develop through the nucleation process with a relatively high surface energy barrier, and their formation rate is solely controlled by the release rate of Ag^+^ from the wire, thus by the applied current magnitude. The Ag/AgCl electrode with a thick AgCl layer and many holes in the AgCl surface structure like microchannels is considered to work effectively for the LFEFD sensor in terms of both detection sensitivity and service lifetime.

## Introduction

The Ag/AgCl electrode is one of the most widely used electrodes.^1−4^ Its applications range from its use in the reference electrode,^2,5,6^ through as the sensor determining the chloride content in reinforced concrete structures to assess the probability of chloride-induced corrosion of steel reinforcements in them,^3,7−10^ to the detection of electric field signals in seawater.^4,11−13^ In particular, the Ag/AgCl electrode with AgCl coatings on the Ag surface is robust and nonpolarizable and has a single electric conductive ion, Cl^–^ and establishes the fast electrochemical equilibrium with Cl^–^ containing seawater.^12^ As a result of these properties, it has lower resistance and impedance with the stability of electrode voltage, that is, a very small self-noise in seawater, making itself suitable for its use of the marine weak low-frequency electric field detection (LFEFD), compared to other electrodes such as zinc, graphite, and saturated calomel electrodes, and even better than carbon fiber electrodes.^12^

The common methods to fabricate the Ag/AgCl electrode include the electrolytic process,^1,3,13,14^ hot dipping process,^15^ and sintering process.^16^ Among them, the electrolytic process in which constant current (i.e., chronopotentiometry) or potential (i.e., chronoamperometry) is applied has the Ag electrode connected to the working electrode probe of the work station and anodized in the chloride-containing such solutions like HCl, KCl, and NaCl to form the AgCl layers on the electrode surface. It is cost-effective and exhibits good stability and a large surface area.^4^

It has been reported that the electrochemical performance of the Ag/AgCl electrode fabricated by the electrolytic process is greatly influenced by the structure on the surface of the electrode,^3,9,14^ emphasizing the importance of controlling the surface structure and deep understanding of the AgCl formation mechanism on the Ag surface. Because of this reason, numerous investigations^3,17−24^ on the AgCl formation on the Ag surface have been conducted in the chloride-containing solution by hiring methods such as linear sweep voltammetry, cyclic voltammetry (CV) tests, potentiostatic current-time transient experiments, and potentiodynamic polarization tests.^3,17,18^ They suggested that the AgCl layers form by the AgCl nuclei formation on the Ag surface followed by their growth which is controlled by both interfacial and diffusion crystal growth kinetics.^3,17^ The theoretical interpretation of such CV curves in these studies provided invaluable information to understand the AgCl formation, including the indications of the nucleation and growth process involved in it and the aforementioned growth kinetics. However, the morphological demonstration of the evolution of surface structures on the Ag surface, linking them with the specific stages in CV curves or curves of potential (or current) vs anodization time, is still scarce.

In this study, we aim to address this lack of knowledge in the details of the morphological evolution on the Ag surface for the AgCl formation and then propose an optimal AgCl structure on the electrode surface for the actual application to the LFEFD Ag/AgCl sensor. Thus, we have used the wire with a dimension of 2 mm × 11 cm, which is the size of the Ag/AgCl electrode designed for actual applications in the LFEFD in seawater. To reveal the AgCl formation linked with specific stages in the anodization process, we have tracked the morphological changes occurring on the Ag wire surface in 3.3 M KCl solution from the time of early anodization by the constant current method to later time via scanning electron microscopy (SEM)/energy-dispersive X-ray spectroscopy (EDX) and X-ray diffraction (XRD), together with complementary analyses including the analysis of the induction time in the anodization curves and an assay of Cl^–^ concentration nearby the wire as a function of anodization time by ion chromatography (IC).

Our results show that at the beginning of anodization where potential little increases with time in the anodization graph, dissolution of Ag from the wire surface into the anodizing solution dominates, although a certain level of AgCl formation occurs. Then, with the abrupt potential rise, the main AgCl formation event, accompanied by the formation of the assumed thin AgCl deposits covering a large portion of the wire surface, proceeds. These deposits with no clear facet planes grow next to one another keeping this interfacial structure and become individual deposits in sizes of about several micrometers to 10 μm connected to themselves and form the network structure, covering the wire surface. This represents the main developing mode of the AgCl deposits. We assess the magnitude of the nucleation barrier of the AgCl deposits by applying a classical nucleation theory to the anodization graphs and also the rate-determining stage for the formation by the analyses through XRD and IC measurements, providing an insight into the visual data of the AgCl deposits.

Then, the visual data of AgCl structures are linked with the resistance values at 1 Hz—an important parameter for the electrochemical performance of the LFEFD sensor electrodes—from the electrochemical impedance measurements, and the desired AgCl structure on the Ag wire surface is proposed for the actual application to the LFEFD Ag/AgCl sensor electrode in seawater.

## Results and Discussion

### Anodization Process

**Figure 1:**
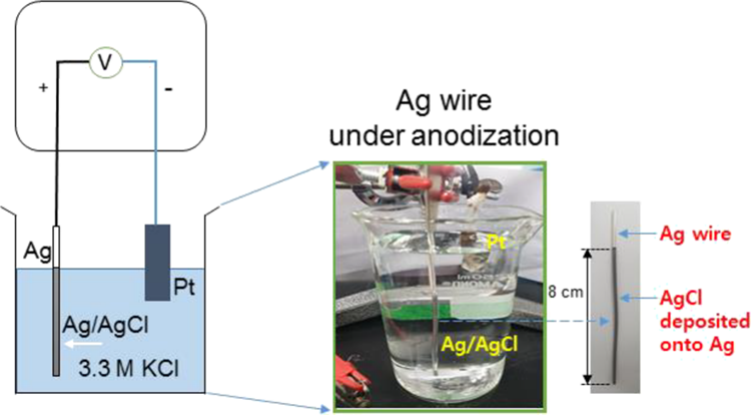
Schematic of the anodization of Ag wire using the two-electrode setup where the wire (2 mm (diameter)) is connected to the working electrode probe and platinum (Pt) foil to the counter electrode probe under the constant current conditions (see the Experimental Section for details.).

Anodization of Ag in the chloride-containing solutions such as NaCl,^13^ HCl,^3^ and KCl^19^ solutions converts a portion of the Ag surface to AgCl. Figure 1 shows our setup of the anodization of the Ag wire using the two-electrode system; note we also conducted experiments in the three-electrode setup in which we obtained potential (V vs Ag/AgCl) against the Ag/AgCl reference electrode to convert it into potential (V vs RHE) against a reversible hydrogen electrode. The Ag wire had a diameter of 2 mm and a length of about 11 and 8 cm of its length—on which surface the AgCl formed—was immersed in the 3.3 M KCl solution (250 mL).

### Morphological Evolution on the Ag Wire Surface for the AgCl Formation during Anodization at a Constant Current of 1 mA (0.2 mA/cm^2^) for 1 h

**Figure 2:**
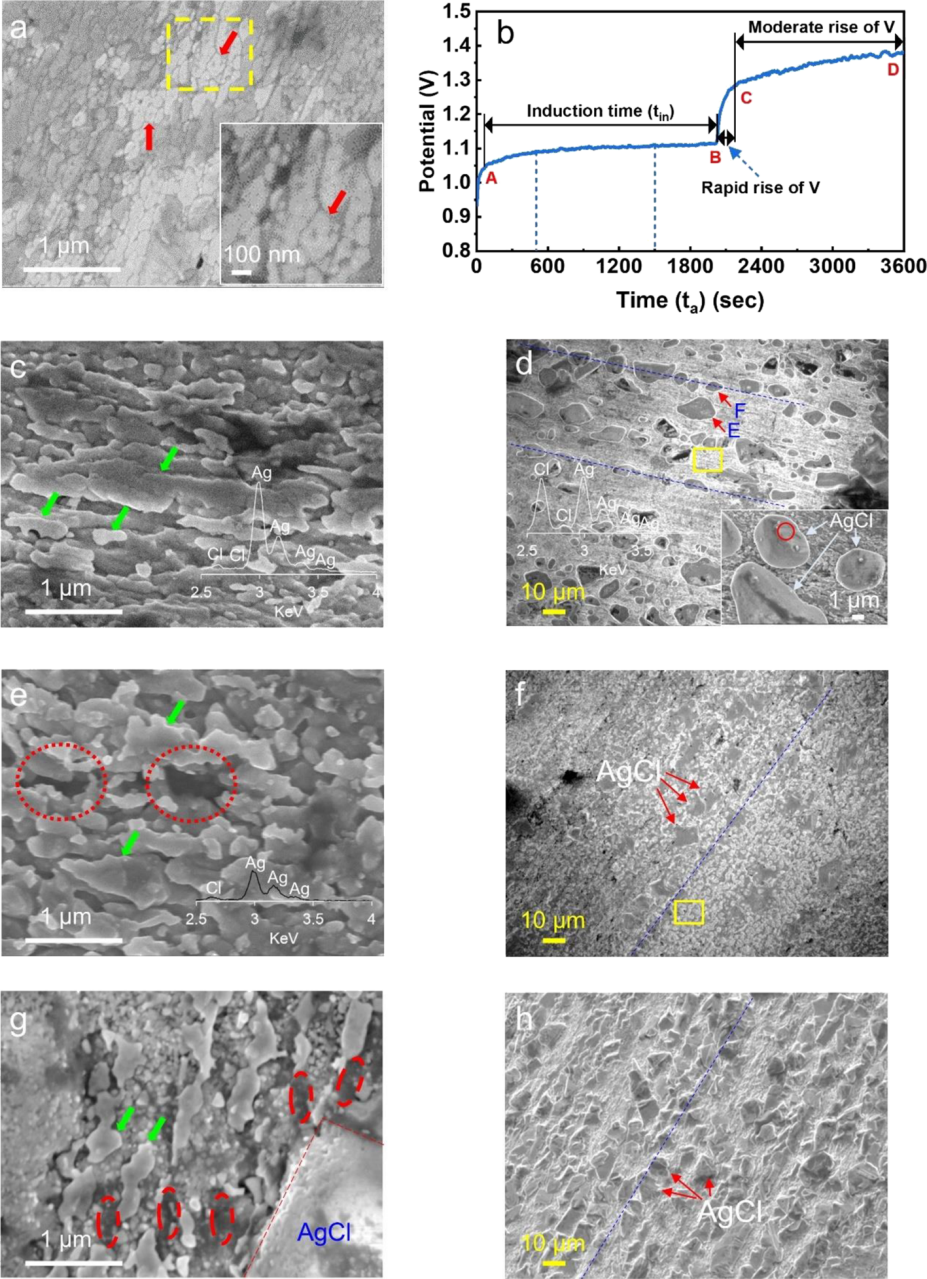
SEM images of the morphological changes on the Ag wire surface during anodization under a constant current of 1 mA as a function of anodization time and EDX analysis. (a) Surface of the Ag wire prior to anodization. (b) Graph of the anodization of the wire at the constant current of 1 mA (0.2 mA/cm^2^) for 1 h (i.e., 3600 s). (c–h) Surfaces of the wire corresponding to specific times of the graph of anodization displayed in (b) where (c), (d, e), (f, g), and (h) are for 500, 1500, 2310, and 3600 s. The embedded EDX spectra in (c), (d), and (e) display elemental compositions (atomic %) of the whole surface shown in (c), such particles in the inset of (d), and the whole surface shown in (e), respectively.

The surface of the Ag wire before being applied to anodization is shown in Figure 2a at high resolution (see Figure S1 for the SEM images of more Ag surface areas).

This image served as a reference from which the morphological changes occurring on the Ag surface during anodization under the constant current were discerned.

As seen, the surface is composed of small grains, such as ones indicated by thick red arrows, overall in sizes of several tens of nanometers to about 100 nm, without specially distinct features on it (see the inset for the magnified view of the area inside a dashed yellow box).

To track the morphological changes occurring on the Ag surface, including the AgCl formation in a manageable way, we made use of examining the Ag surface exposed to a mild anodization condition: for our case, 1 mA (i.e., 0.2 mA/cm^2^) was applied to the Ag wire immersed in 3.3 M KCl solution. This mild condition rendered us to track the process of morphological changes of the surface of the wire manageably so that the overall evolution of the process could be understood clearly, although the data analysis was performed with ex situ SEM data, not in situ. Figure 2b displays the typical graph of the anodization of the Ag wire using the setup shown in Figure 1 at a constant current of 1 mA, displaying potential (*V*) versus the anodization time (*t*_a_). Upon onset of the anodization process, *V* rapidly rises up to an average of 1.09 V (±0.03 V) and then, typically, very slightly increases over a long period of the average 2303 s (±242 s) to 1.12 V (±0.03 V), with almost stagnation of its rising during this period; note that each time there existed some variations in the length of the induction time (*t*_in_), although the same procedure was applied. However, the evolutionary shape of the anodization curves was the same as that seen in Figure 2b (see Figure S2 for several anodization curves under a constant current of 1 mA). Although the potential increase is very small, the surface of the Ag wire was found to have experienced a large morphological change, which was evaluated by the SEM and EDX analyses. Figure 2c shows the Ag wire surface upon anodization for 500 s. As seen by a comparison of grains indicated by green arrows in Figure 2c with those by red arrows in Figure 2a, the Ag surface initially with grains overall in sizes of several tens of nanometers to about 100 nm in Figure 2a now exhibits larger and elongated grains in Figure 2c, totally changed compared with its initial surface (Figure 2a) before anodization. The inset EDX spectra (∼0–1% Ag) of the surface of Figure 2c show that the surface is Ag, indicating that these grains are not AgCl. Thus, the SEM image together with these EDX data could indicate that the area of the Ag grain boundary decreases by the grain growth by the migration of Ag atoms or ions from small grains to larger grains to reduce the total surface energy while the dissolution of Ag happens on the wire surface.

As the anodization proceeded further, more morphological changes on the wire surface were observed. Figure 2d shows the wire surface anodized for 1500 s. As seen, deposits or particles with kinds of polygonal shapes or crystal planes overall in sizes of several micrometers to about 10 μm such as ones indicated by red arrows labeled with E and F formed. The inset image at a higher magnification shows the morphology of such particles in detail and they are AgCl particles as proved by the EDX spectrum embedded in the image; the spectrum represents the atomic composition of the elements of a spot (such as a red circle) on a single AgCl deposit.

For the early stage of the electrolytic process of AgCl formation on the Ag surface, the surface structure of bare Ag such as scratch—presumed energetically favorable site—plays an important role (see Figure S1 for the bare Ag surface with scratch lines).^14^ As seen by those AgCl deposits located along dotted blue lines in Figures 2d,f,h and S3 (more clearly shown) and in Figure 2d of the paper of Ha and Payer,^14^ the AgCl deposits with crystal planes preferentially form along with the scratch line directions on the Ag surface. As reported in many studies,^3,14,18^ they form by the nucleation and growth process. Given that, commensurate with the suggestion by Ha and Payer, their formation along the scratch lines on the Ag surface could be due to the reduced surface energy barrier for nucleation at these heterogeneous sites compared to other surface areas or the increased Ag^+^ concentration leading to higher supersaturation at the scratch bottoms.

Chemically, the AgCl deposits form on the silver wire surface while silver is oxidized in the 3.3 M KCl solution during anodization by the following net reaction:^3,4,19^
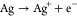
1
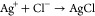
2

Although the AgCl formation occurs simply by the combination of the dissolved Ag^+^ and Cl^–^ by the net reaction described by eqs 1 and 2, it is known that AgCl does not form by direct combination of Ag^+^ and Cl^–^, rather through the transformation of soluble species AgCl_*n* + 1_^–*n*^ (0 ≤ *n* ≤ 3) first formed from the combination of the Ag^+^ and Cl^–^ into the solid AgCl phase.^19^

Figure 2e shows the details of the surface area like that seen within a yellow box in Figure 2d which is near the AgCl deposits. The surface morphology changed further as the anodization proceeded when compared to that in Figure 2c. Overall the grains (indicated by green arrows) rather became larger and cavities such as those within dotted red circles, formed by the dissolution of Ag surface, were observed. The inset EDX spectrum (Ag:Cl = 94.3:5.7 (at%)) shows that the surface shown in Figure 2e is still representatively Ag. Thus, these grains are Ag, although some morphological changes happened compared with those in Figure 2c. Therefore, Figure 2a,c,e demonstrate that the Ag dissolution is accompanied by the morphological changes in the Ag grain.

As the anodization proceeded further, the very slight potential increase was followed until the end time point (B in Figure 2b) of the induction period; although the potential increase was small, the surface nearby the AgCl deposits was found to experience the morphological changes continuously. Then, the potential rose rapidly over a short period of about 100 to 200 s, as seen by the time point C in Figure 2b. Figure 2f shows a surface corresponding to the time point C. When this surface at this stage is compared with the surface of Figure 2d at the previous stage, the most striking difference lies in the morphology and composition of the surface nearby the AgCl deposits. Figure 2g shows such a surface like that seen within a yellow box in Figure 2f in a high resolution and it differs a lot from those seen in Figure 2c,e. The EDX spot analyses (∼1–2 at% Cl) of grains like those indicated by thick green arrows in Figure 2g indicated that they are Ag (see also Ag grains indicated by thick green arrows in Figure 2c,e). Somewhat dark areas—such as those marked by dotted red ovals—that seem to make an embedded continuous network structure and represent the characteristics of a large portion of the surface at this stage showed ∼29 at% Cl content by the EDX spot analyses. The measurements on these areas were applied for a very brief moment in an effort to obtain the elemental information about the only targeted spots. Most likely, these dark areas represent thin AgCl deposits. This is because they exhibit ∼29 at% Cl content and their formation is overall related to the sudden potential increase, that is, rise in resistance in the anodization curve which implies a sudden increase in the deposited amount of the insulating AgCl. The latter is also supported by XRD data dealt with later.

Starting from the time point C in Figure 2b, it is followed by a moderate potential increase up to the end time point D of the process of 1 h anodization at 1 mA. Figure 2h shows a surface anodized for 1 h at 1 mA. The AgCl deposits in sizes of several micrometers to about 10 μm seem to occupy more surface than those observed in Figure 2f; the relative amount of formed AgCl at specific anodization times can be assessed more quantitatively by the XRD data shown later than these SEM data because the SEM observation is conducted on local locations, although they are strong in that they provide visual data.

### Morphological Evolution by Further Anodization at 1 mA (0.2 mA/cm^2^)

**Figure 3:**
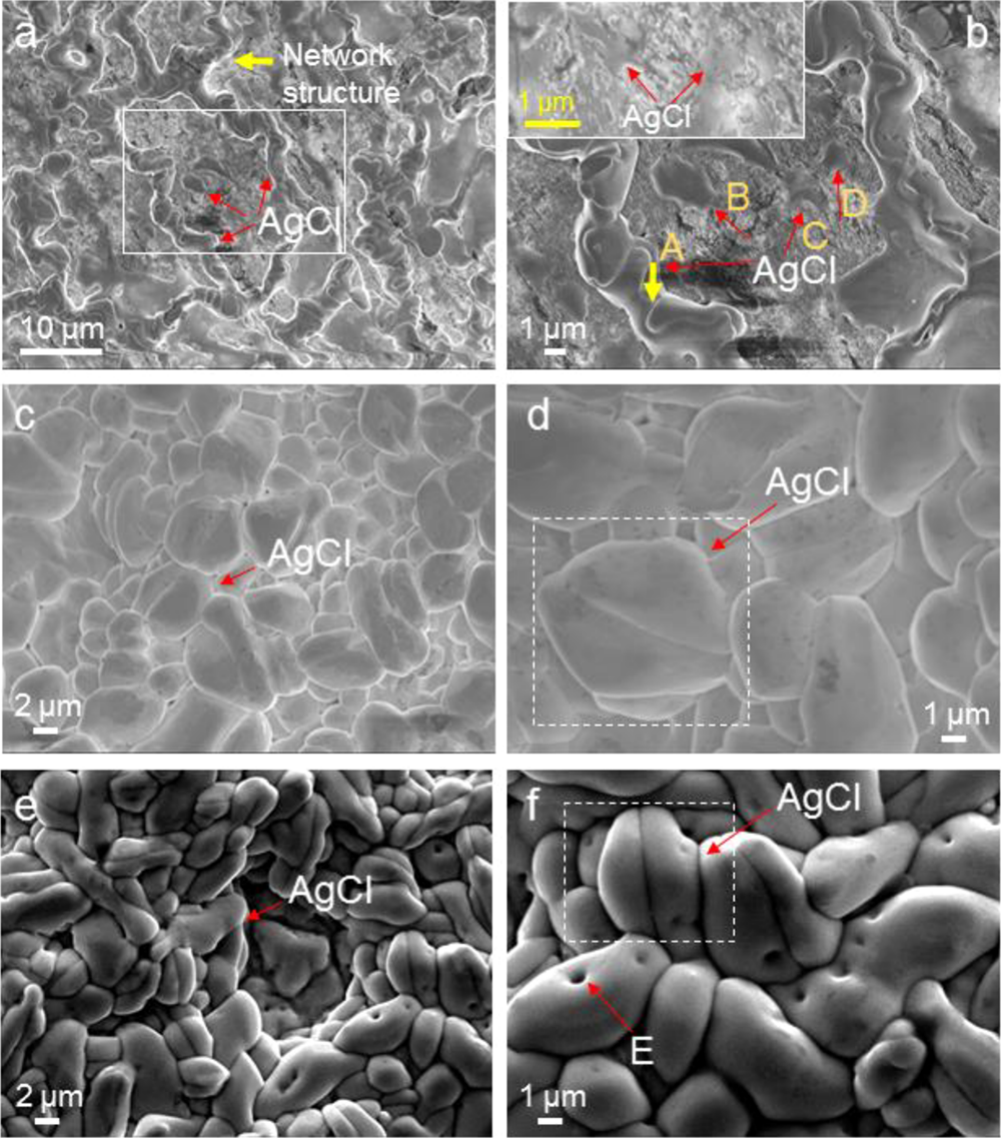
SEM images of the morphological changes on the Ag wire surface during anodization at (a, b) 1 mA (0.2 mA/cm^2^) for 2 h, (c, d) 1 mA for 4 h, and (e, f) 4 mA (1.6 mA/cm^2^) for 1 h.

As the wire was anodized further, more amount of AgCl was observed to form on the wire surface. Figure 3a shows a surface anodized at 1 mA for 2 h. The AgCl deposits seem to have formed a network. As seen by a thick yellow arrow “A” in Figure 3b which represents the area within a white box in Figure 3a, the network has boundaries in it, suggesting that it is composed of AgCl deposits individually nucleated and grown. Careful examination of SEM images suggests how this network was derived. It shows the individual AgCl deposits indicated by B, C, and D stemmed from such locations indicated by dotted red ovals in Figure 2g which showed ∼29 at% Cl content by the EDX spot analyses. The inset image at a higher magnification supports further this suggestion, as judged by thin deposits (indicated by red arrows) covering the surface, that they originated from the locations indicated by the dotted red ovals in Figure 2g. Then they grew to be deposits overall in sizes of several micrometers to about 10 μm next to one another, connected together to form the network structure. Interestingly when these network structures dominated, the AgCl deposits with the clear facet planes shown in Figure 2h were not observed; it was reported by an earlier study^19^ that the deposits with these well-defined crystal planes were rarely observed. This could suggest that the deposits with the facet planes might be dissolved and Ag^+^ and Cl^–^ formed by this dissolution join the deposits with no clear crystal planes when they grew to make the network structure.

As the anodization proceeded at 1 mA for 4 h, the surface was observed to be fully covered by AgCl by SEM observation as seen in Figure 3c,d, although the SEM could not confirm that all the surface was completely covered by the AgCl, given that it probed the area locally. Interestingly, twined AgCl deposits or particles like one as shown in a dotted white box in Figure 3d were observed. As seen in this one, the AgCl deposits also formed on the already formed ones, with the full coverage.

### Comparison of the Structures of AgCl Deposits Fully Covering the Ag Wire Surface by the Applied Current Magnitude

The very similar AgCl structure that formed under the condition of 1 mA was observed on the wire surface by the anodization under the constant current conditions of other current values (2 (0.4), 4 (0.8), and 8 mA (i.e., 1.6 mA/cm^2^)). For example, Figure 3e,f shows the surface structure on the wire anodized at a constant current of 4 mA for 1 h— theoretically by Faraday’s first law (Discussion S1);^3^ the condition of 4 mA for 1 h has the same thickness or amount of deposited AgCl as the condition of 1 mA for 4 h. The surface structure is very similar to that shown in Figure 3c,d, with the full coverage of AgCl and twined AgCl deposits. Also, microchannels such as one indicated by “E” running through the particles are observed, as reported in others’ research.^3,14^ They are considered the main path for ionic transport for thick AgCl layers, contributing to the formation of the multiple AgCl deposits.^14^

The difference between the two conditions seems to lie in the overall individual AgCl deposit sizes. Seen qualitatively by a comparison of Figure 3d (1 mA for 4 h) with Figure 3f (4 mA for 1 h), the individual deposits in Figure 3d look somewhat larger than in Figure 3f. It is reasonable given that the 1 mA condition provides a 4-fold smaller number of Ag ions to the solution next to the Ag wire than the 4 mA condition for a given time and thus induces less supersaturation next to the wire while the Ag ions also diffuse away. This will result in larger nucleus sizes that may lead to larger deposits. However, the difference in the AgCl deposit sizes in the fully developed AgCl structure is observed to be small, as mentioned above. Thus, under the condition of current density values at least less than 0.8 mA/cm^2^ (i.e., 4 mA condition), the fully developed AgCl structure with the full coverage of Ag wire is controlled by the current magnitude (or density) multiplied by anodization time.

### XRD Analysis of AgCl Formation

**Figure 4:**
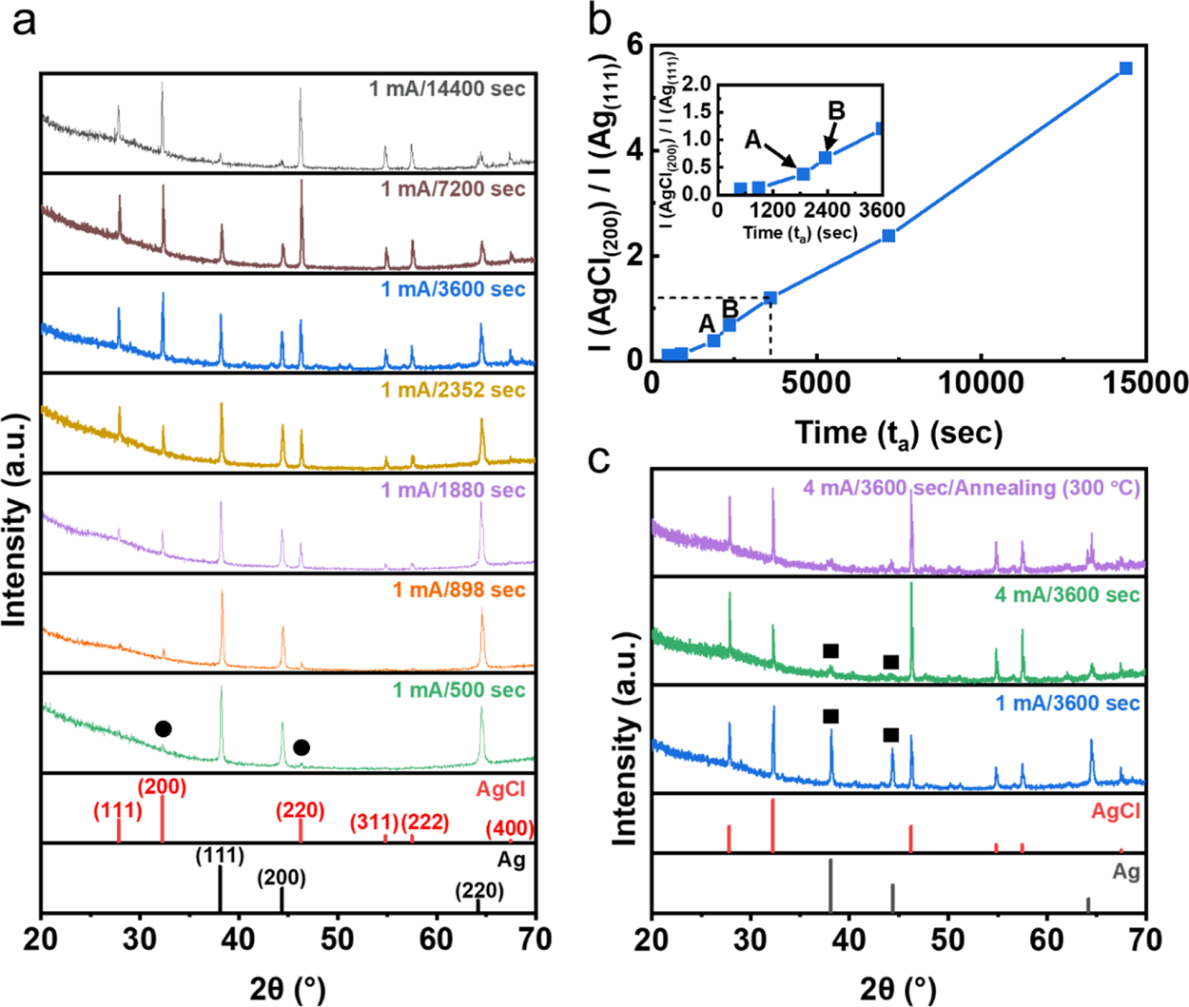
XRD patterns of the Ag wire by anodization under constant current conditions. (a) Ag wire as a function of anodization time (*t*_a_) up to 14,400 s under a constant current of 1 mA. Reference peaks of Ag (black color) and AgCl (red color) are displayed at the bottom of the graph. The times 1880 and 2352 s correspond to times just before the occurrence (point B) of the abrupt potential rise and such point like C just after the ending of the abrupt potential rise in Figure 2b, respectively. (b) Ratio of the diffraction peak intensity (*I*) of (200) AgCl to (111) Ag. The (200) AgCl and (111) Ag peak intensities are selected to demonstrate the evolution of AgCl formation with anodization time at 1 mA. The inset magnifies the part of the graph in a dotted box. (c) Ag wires anodized at a constant current of 1 and 4 mA for 1 h and Ag wire anodized at 4 mA for 1 h followed by annealing at 300 °C for 1 h.

The evolution of the amount of AgCl deposits formed on the Ag wire with anodization time (*t*_a_) was estimated through the XRD analysis. Figure 4a shows the XRD patterns of the Ag wire anodized at a constant current of 1 mA as a function of *t*_a_ up to 14,400 s (i.e., 4 h).

As is shown, as *t*_a_ increases, the peak intensity (*I*) of AgCl increases while *I* of Ag decreases, indicating the increase of the formed AgCl amount with the increase of *t*_a_. It is noteworthy that the very weak peak intensities (black circles) of (200)AgCl and (220)AgCl at the *t*_a_ of 500 s are manifested, suggesting the formation of AgCl. The discrepancy between these XRD results and SEM observation in which we did not see AgCl deposits or particles in our limited search is probably due to the nature of SEM observation which probed local areas.

To show the effect of *t*_a_ on the evolution of the formed AgCl amount at 1 mA concisely, we compare *I* of the representative AgCl and Ag peaks, (200) AgCl and (111) Ag peaks, as seen in Figure 4b. As the anodization proceeds, initially *I*(200)AgCl/*I*(111)AgCl little increases and then, overall, it increases proportionally to *t*_a_. This suggests that the amount of AgCl increases at a constant rate with *t*_a_ up to 4 h at 1 mA and implies that the release rate of Ag^+^ determines the AgCl formation rate. Also, note that the increase of the intensity ratio between time point A (1880 s) and B (2352 s) may support that such locations indicated by dotted red ovals in Figure 2g which showed ∼29 at% Cl content by the EDX spot measurements are thin AgCl deposits mostly formed during the abrupt potential rise manifested between the time points B and C in Figure 2b.

Figure 4c shows the XRD patterns of the Ag wire anodized at a constant current of 1 or 4 mA for 1 h and of the wire anodized at 4 mA for 1 h followed by annealing at 300 °C for 1 h. A comparison of the XRD patterns between anodization at 1 mA for 1 h and anodization at 4 mA for 1 h shows the effect of the applied current magnitude on the formed AgCl amount. While (111) and (200) Ag peaks (black rectangles) are manifested in some magnitude for the Ag wire anodized at 1 mA for 1 h (blue), they are little expressed for the wire anodized at 4 mA for 1 h (green), because the higher current of 4 mA for 1 h formed a larger amount of AgCl than 1 mA for 1 h. These XRD results are commensurate with the results from the SEM images where full coverage of AgCl deposits or particles (Figure 3e,f), mostly in a size of several μm, was observed for 4 mA with the anodization of 1 h while a partial coverage of them (Figure 2h) was manifested for 1 mA with the anodization of 1 h. When the XRD pattern of anodization at 4 mA for 1 h is compared with that from the annealed one (300 °C, 1 h), little difference is observed. This may suggest that the degree of crystallinity of AgCl deposits is very similar between the as-prepared and the annealed one and that the AgCl deposits formed by the anodization have already well-defined crystalline structures.

### Estimation of the AgCl Surface Energy from the Analysis of Induction Time Influenced by the Applied Current Magnitude

The magnitude of the applied current controls the amount of Ag^+^ released from the Ag wire surface for a given time, that is, the Ag^+^ release rate from the wire surface during anodization. Because the AgCl formation occurs by the nucleation and growth process as reported in many studies,^3,14,18^ the magnitude of the applied current directly controls the AgCl formation by influencing supersaturation (σ) that drives the nucleation and growth process.

**Figure 5:**
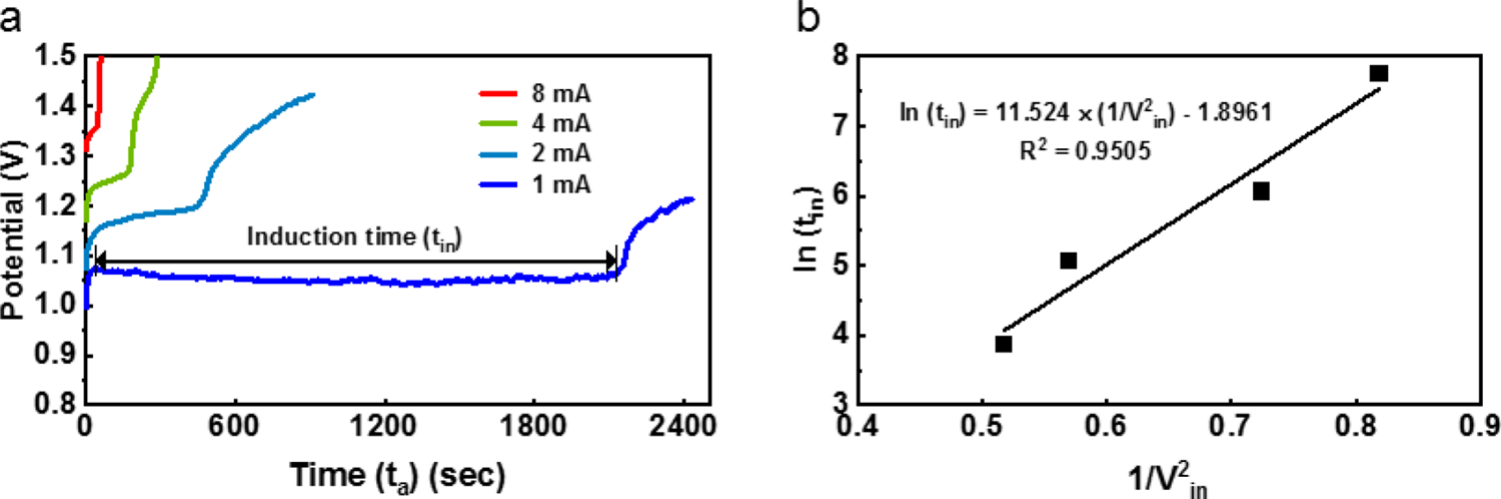
Induction time (*t*_in_) as a function of the applied current (a) and the relationship (b) between ln (*t*_in_) and 1/*V*^2^_in_ where *V*_in_ is the average *V* during the induction time.

As already seen in Figure 2b, the potential has the stagnation of its rising, that is, induction time (*t*_in_) in a course of anodization at a constant current of 1 mA. This shape of the curve with an initial induction time (or lag phase) before an abrupt signal increase is typical of graphs of signal intensity vs time observed in many systems having the nucleation process for solid formation which range from inorganic^24−27^ to organic^28^ and biological systems.^29^ In these cases of the nucleation processes, as σ increases, *t*_in_ is reduced. Because σ scales with the applied potential (*E*) by the following eq 3,^30^*t*_in_ is expected to be reduced with the increase of *E* which is rendered by the increase of the applied current.
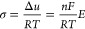
3where Δ*u* is the chemical potential change accompanied when a mole of reaction product forms, *R* is the gas constant (8.314 J/Kmol), *T* is the absolute temperature (K), *n* is the number of electrons transferred for the reaction (for this case of Ag, *n* = 1), and *F* is Faraday’s constant (*F* = 96,500 C/mol/equiv). As expected, *t*_in_ was reduced when the applied current increased as displayed in Figure 5a (see also Table 1 for *t*_in_ for the different applied current magnitudes and the average *V*_in_ for each induction time).

**Table 1:** Average Potential (*V*_in_) for Each Induction Time (*t*_in_) Corresponding to the Magnitude of the Applied Currents (*I*)

*I* (mA)	*V*_in_ (V)	*T*_in_ (s)
1	1.11 ± 0.03	2303 ± 242
2	1.18 ± 0.10	428 ± 82
4	1.33 ± 0.09	159 ± 34
8	1.39 ± 0.07	47 ± 7

Then, taking the primary nucleation theory, *t*_in_ is related to σ by the following correlation:^26^
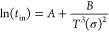
4
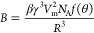
where A is a dimensionless empirical constant, β is the shape factor, γ is surface energy (J/m^2^), *V*_m_ is the molar volume (m^3^/mol) for solids, *N*_A_ is the Avogadro’s number (6.022 × 10^23^/mol), *f*(θ) is a correction factor which is 1 for purely homogeneous nucleation and 0.01 for heterogeneous nucleation, and other symbols are already defined in eq 3. Following eq 3, eq 4 can be expressed by substituting  for σ as
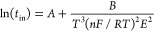
5

Thus, when AgCl is formed via nucleation, a plotting of ln(*t*_in_) versus (1/*E*^2^) should give a straight line with a slope of . As expected, the plotting manifests the straight line (Figure 5b). Then, if β and *f*(θ) are known or assumed in *B*, γ can be obtained from the slope. By taking β = 16π/3 (assuming the spherical AgCl nucleus),^26^*f*(θ) = 0.01 (because AgCl forms on the Ag surface), and values of other known parameters (*V*_m_ = 6.612 × 10^–10^ m^3^/mol, *N*_A_ = 6.022 × 10^23^/mol, *T* = 298 K, *n* = 1, *F* = 96,500 C/mol, and *R* = 8.314 J/Kmol), and the obtained slope of 11.524 J^2^/C^2^, we get γ = 1.586 J/m^2^ (=1586 erg/cm^2^).

The obtained value of γ is about two times higher than grain boundary energy (756 erg/cm^2^) of gamma-phase iron^31^ at 1350 °C and several times higher than the reported surface energy of such inorganic substances like calcite (CaCO_3_, 218 erg/cm^2^ for {10.4}^32^) and sodium chloride (NaCl, 161 erg/cm^2^ for (100)^32^) and much greater than the surface energy of calcium oxalate monohydrate (CaC_2_O_4_·H_2_O, 7.14 erg/cm^2^) crystals.^26^ This suggests that the AgCl nucleation at the Ag substrate is a process with a relatively high energy barrier.

### Rate-Determining Factor for the AgCl Formation by Anodization in 3.3 M KCl Solution under a Constant Current of 1 mA

When assuming that the main AgCl formation via the nucleation process starts at the end of induction time (*t*_in_), the chemical potential change (Δ*u*) when a mole of AgCl forms via nucleation can be obtained by eq 6 in consideration of the net AgCl formation reaction, if the concentrations of dissolved Ag^+^ and Cl^–^ near the Ag wire at the end of *t*_in_ are known:
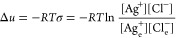
6where, as aforementioned, *R* is the gas constant, *T* is the absolute temperature, σ is supersaturation, and [Ag^+^] ([Ag_e_^+^]) and [Cl^–^] ([Cl_e_^–^]) are the actual and equilibrium silver (chloride) ion concentrations in the 3.3 M KCl solution.

Obtaining [Ag^+^] near the Ag wire surface in a course of anodization was not possible because the substantial dilution of the anodizing solution to drop KCl weight percentage below 5% required for inductively coupled plasma measurements placed [Ag^+^] below the detection limit; the measurements of [Ag^+^] in the anodizing solution as a function of anodizing time were conducted unsuccessfully due to the described reason. However, [Cl^–^] with time during anodization could be successfully measured because the initial concentration of 3.3 M in the anodizing solution put [Cl^–^] at a concentration level enough for detection for even IC measurements (see the Experimental Section for the procedure).

**Figure 6:**
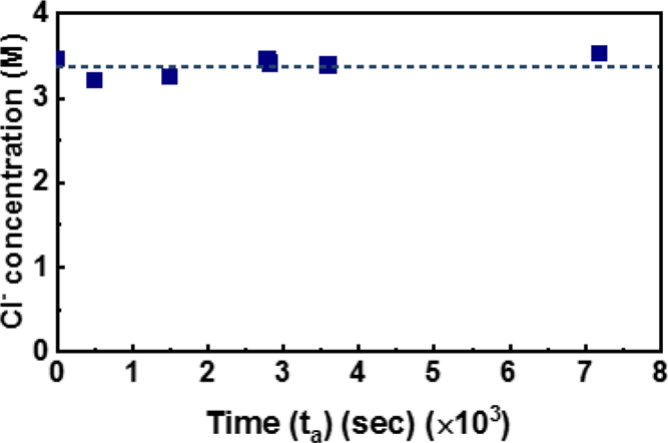
Evolution of the concentration of Cl^–^ ([Cl^–^]) near the Ag wire during anodization at a constant current of 1 mA.

Figure 6 shows the evolution of [Cl^–^] near the Ag wire during anodization at a constant current of 1 mA.

The anodization times, 3600 and 7200 s, are times that form a theoretical AgCl thickness of 1.9 and 3.8 μm, respectively, by Faraday’s first law (see Discussion S1). As seen, there was constant Cl^–^ concentration as the same as the value of the initial concentration (3.3 M) of the anodizing solution up to measured 7200 s. Also, [Cl_e_^–^] for the solid AgCl formation in this 3.3 M KCl solution during anodization can be taken the same as [Cl^–^] of 3.3 M because solid AgCl is dissolved so tiny in the 3.3 M KCl solution of 250 mL that [Cl^–^] of 3.3 M is kept. Then eq 6 is expressed as:
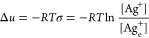
7

This suggests that the concentration of Ag^+^ near the wire surface released by anodization solely controls Δ*u* needed for the formation AgCl via nucleation in this anodizing condition of 3.3 M KCl solution. Because the release rate of Ag^+^ from the wire surface, that is, the increase rate of Ag^+^ concentration near the Ag surface, increases as the applied current magnitude increases, the length of the induction time needed to reach the required value of Δ*u* for the formation of AgCl was reduced with the higher applied current magnitude (see Figure 5a).

By eq 3, Δ*u* connected to the Nernst equation is also expressed as the following:
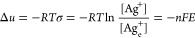
8

To obtain Δ*u* at the end of the induction time by this eq 8, anodization by the three-electrode setup that uses a standard Ag/AgCl electrode as the reference electrode and platinum foil as the counter electrode was conducted under the constant current condition of 1 mA (see Figure S4). Upon inserting *n* = 1, *F* = 96,500 C/mol/equiv, and *E* = 0.75 V vs RHE (converted from the *E* measured against the Ag/AgCl reference electrode as seen in Figure S4) into eq 8, we get Δ*u*= −72.38 kJ/mol. Then, with *R* = 8.314 J/Kmol and *T* = 298 K, we obtain σ = 29.2. This value of supersaturation (σ = 29.2) is much higher than values in which nucleation occurs in many other crystallizing systems such as inorganic calcite (CaCO_3_, σ > 3.5)^33^ and calcium oxalate monohydrate (CaC_2_O_4_·H_2_O, σ > ∼2).^26^ It is also in good agreement with the obtained relatively high surface energy (1586 erg/cm^2^) value of AgCl compared to the aforementioned values of calcite (218 erg/cm^2^) and COM (7.14 erg/cm^2^) because higher supersaturation is needed for nucleation in the system where a higher surface energy barrier is involved.

### Insight into the Desired Surface Structure of the Ag/AgCl Electrode for the Application to the LFEFD Sensor

The sensor electrodes used for the LFEFD in the marine environment have a high demand for having low resistance values at low-frequency signals for high detection sensitivity.^4^ Thus, the electrode resistance values at 1 Hz under sine wave excitation signals of 5 or 10 mV are widely used to represent an important parameter of the electrochemical performance of the sensor electrodes.^4,12^ In this part, we compare the resistance values of the Ag/AgCl electrodes fabricated under conditions of various constant current magnitudes and anodization times and connect them with the surface structure of the electrodes including AgCl deposits.

**Figure 7:**
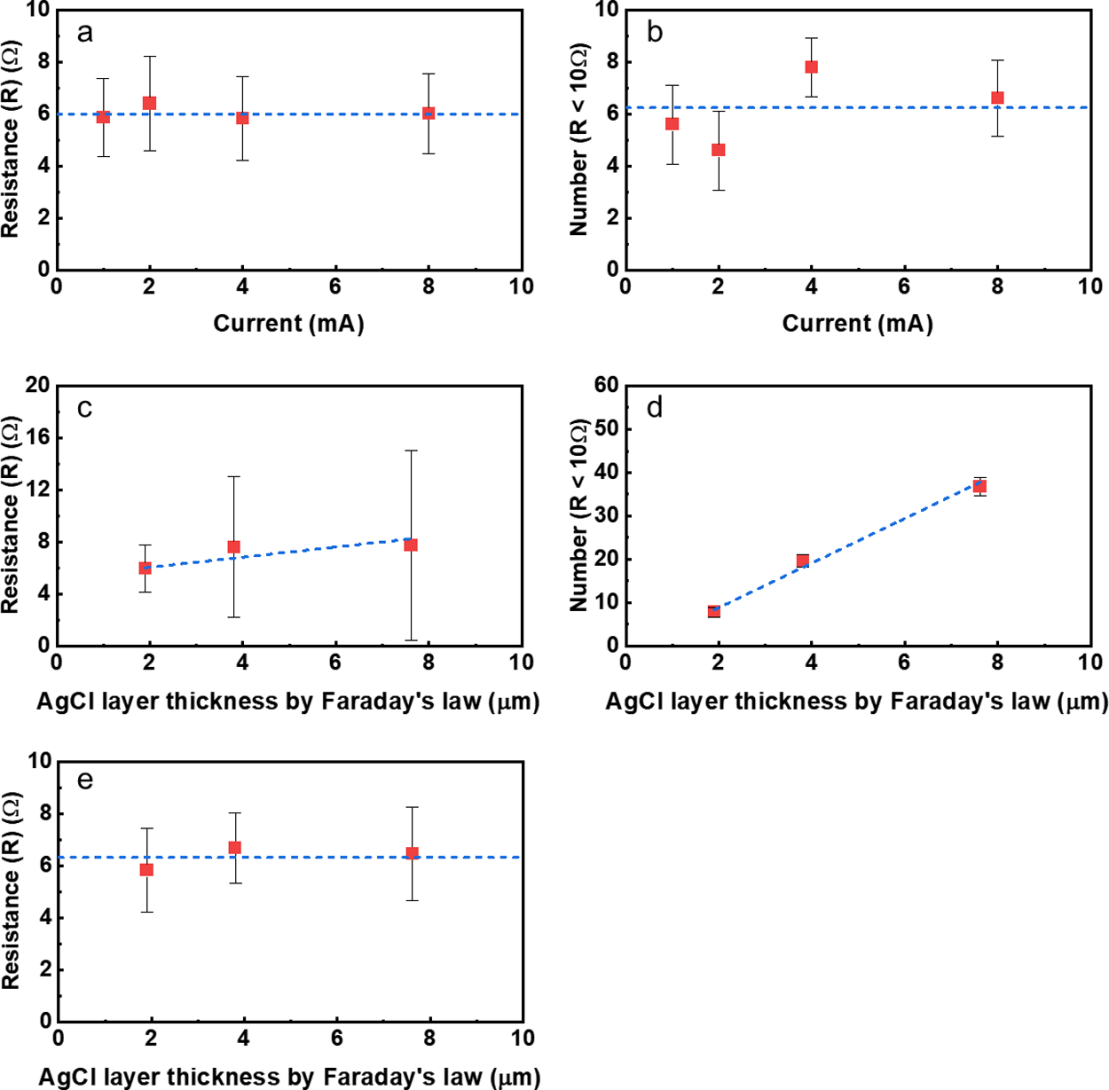
Characterization of electrochemical properties of Ag/AgCl electrodes. (a) Average resistance at 1 Hz of the electrodes fabricated under constant currents of 1 mA for 3600 s, 2 mA for 1800 s, 4 mA for 900 s, and 8 mA for 450 s. All electrodes have the same theoretical AgCl thickness of 1.9 μm by Faraday’s first law. (b) Number of the repeated impedance measurements recording the resistance value less than 10 Ω at 1 Hz for the anodization conditions specified in (a). (c) Resistance at 1 Hz of the electrodes fabricated under the constant currents of 4 mA with theoretical AgCl thickness up to 7.62 μm by Faraday’s first law. (d) Number of the repeated impedance tests recording the resistance value less than 10 Ω at 1 Hz for the electrodes specified in (c). (e) Average of the repeated impedance tests recording the resistance value less than 10 Ω at 1 Hz for the electrodes specified in (c). The dotted lines in panels (a) to (e) are a guide to the eye.

Figure 7a shows the resistance values (at 1 Hz) of the electrodes fabricated by the anodization under the constant currents of 1 (0.2), 2 (0.4), 4 (0.8), and 8 mA (1.6 mA/cm^2^) that forms theoretically the same AgCl layer thickness of 1.9 μm by Faraday’s first law (see Discussion S1) in 3.5% NaCl solution measured by electrochemical impedance spectroscopy (see the Experimental Section for the measurement method). The values represent the average of the resistance values from the repeated impedance measurements (from five electrode samples for each condition) before resistance exceeds 10 Ω, which is the upper limit of our target resistance values.

As seen, for the same theoretical AgCl thickness of 1.9 μm, the resistance (Avg. about 6 Ω) of the Ag/AgCl electrodes is not affected by the current magnitudes applied to the fabrication of the electrodes. This result is commensurate with that from a previous study^14^ reporting the resistance of the AgCl layer was not dependent on the magnitude of the applied current density under which it grew but on the thickness of the AgCl layer.

Although the anodization conditions for the formation of the 1.9 μm-thick AgCl layer are assumed to have the 1.9 μm-thick AgCl layer cover the whole circumference of the Ag wire when following Faraday’s law, in reality, they do not cover it as that of the expected way, as seen in the SEM image (Figure 2h) and XRD data (Figure 4a) which show Ag peaks for the condition of 1 mA for 3600 s. This could be because the AgCl deposits form individually by the nucleation and growth process and they have either the AgCl thickness larger than 1.9 μm or less than it.

**Figure 8:**
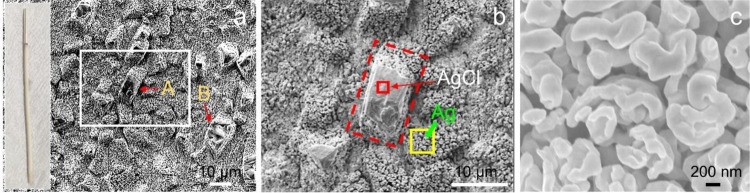
SEM images of the surface of the Ag/AgCl electrode fabricated by the anodization under a constant current of 1 mA for 1 h when resistance just exceeded 10 Ω at 1 Hz after the repeated impedance measurements. (a, b) Surface showing remains of AgCl deposits and Ag. The inset of (a) shows the electrode just exceeding 10 Ω at 1 Hz over the repeated impedance measurements. (b) is the magnified view of the area within the white solid box in (a). The EDX spot analyses of an area of a small red rectangle and of a yellow rectangle showed the ratio of Ag:Cl = 52.85:47.15 indicating that the object is AgCl and Ag:Cl = 98.76:1.24 (at%) proving that the porous surface is Ag. A dotted red rectangle outlines the sides of an originally formed AgCl deposit with the crystal plane by the anodization—many of the AgCl deposits are shown in Figure 2h. (c) Magnified view of an area like that within the yellow rectangle in (b). As seen, now the Ag surface is the porous surface, resulting from the dissolution of the AgCl deposits by the Faradaic reaction occurring during the impedance measurements.

All these electrodes fulfilled the target resistance value (Ω < 10). However, they were considered not to be likely to guarantee the target service lifetime of 2 years as the sensor electrode because their resistance values exceed 10 Ω (Ω > 10) at 1 Hz just after less than 10 times repeats of the impedance measurements as seen in Figure 7b. Because the increased resistance of the electrodes over the measurements should result from their changed surface structures, the SEM measurement was conducted on the surface of the electrode fabricated by the electrolytic process at 1 mA for 1 h which just had Ω > 10 at 1 Hz over the repeated impedance measurements. Figure 8a shows the surface structure of the electrode just exceeding 10 Ω over the repeated impedance measurements; the whole electrode is displayed in the inset. Note that now, macroscopically, the color of the electrode changed from initially dark (Figure 1) to yellow-white. Such objects like those indicated by “*A*” and “*B*” resemble the AgCl deposits shown in Figure 2h. They are proved as the morphologies of those AgCl deposits with crystal planes shown in Figure 2h following the impedance measurements clearly via Figure 8b—the magnified view of the area within a white solid box in Figure 8a—and the EDX spot analyses.

The EDX spot analyses of an area of a small red rectangle and of a yellow rectangle indicated that they are AgCl and Ag, respectively. A magnified view (Figure 8c) of an area like that within the yellow rectangle shows that the Ag surface has the porous structure that resulted from the dissolution of the AgCl deposits which happened by the Faradaic reaction during the impedance measurements.^1^ The importance of this surface observation is that the resistance of the electrode increases when the AgCl deposits are exhausted to a certain level, suggesting that forming a certain large amount of AgCl deposits will be necessary for the long-term service lifetime of the electrode.

Because of this reason, taking the constant current condition of 4 mA, we fabricated electrodes with theoretical AgCl thicknesses ranging from 1.9 to 7.62 μm and measured resistance at 1 Hz which is displayed in Figure 7c. The resistance values represent the average of the resistance values—from the repeated impedance measurements (from five electrode samples for each condition)—summing resistance values from the first measurement with those recorded before resistance again exceeded 10 Ω. As seen, resistance increased with the AgCl thickness. During the measurements, the electrode with an AgCl thickness of 1.9 μm manifested resistance value Ω < 10 from the first measurement except for only one case out of tested five electrodes while electrodes with thicker AgCl thickness showed Ω > 10 (a few tens of Ω) during some initial measurements before beginning to manifest Ω < 10 over the subsequent measurements; the extent of the number of impedance measurements necessary to have Ω < 10 from the first measurement scaled with the AgCl thickness, and this is reflected in Figure 7c where resistance increases with the AgCl thickness.

**Figure 9:**
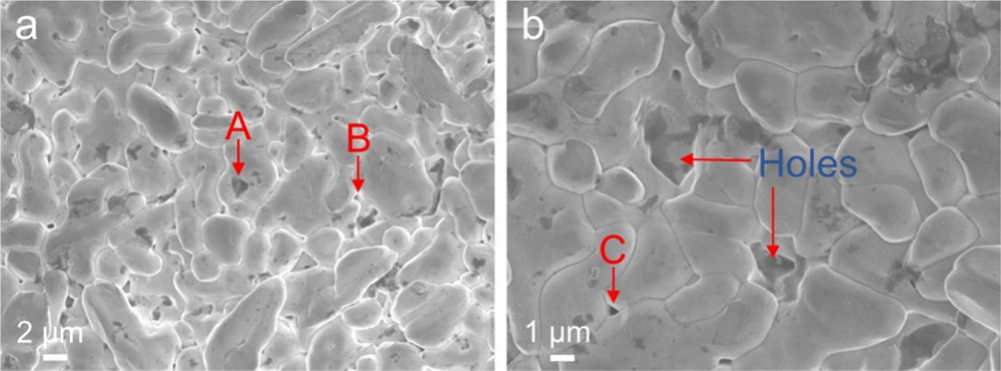
Surface structure of the electrode fabricated under a constant current of 4 mA for 3600 s when it manifested Ω < 10 fifty times over the repeated impedance measurements. (a) Structure of AgCl deposits showing holes (A, B) on it. (b) Structure of AgCl deposits with small (C) and large holes at a higher resolution than that in (a).

There were clear changes in the electrode surface structure when the electrodes manifested Ω < 10, compared to their initial structure. As seen in Figure 9, the electrode fabricated under a constant current of 4 mA for 3600 s when it manifested Ω < 10 fifty times over the continuous impedance measurements shows holes (A, B, C) in the AgCl deposit structure which are many more than microchannels observed in the as-prepared electrode shown in Figure 3f.

These holes are located usually at the boundaries among individual deposits and may form channels leading to the Ag wire surface like microchannels; see Figure 10 of ref (14) for the schematic of microchannels running from the top surface of AgCl deposits to the Ag surface. Because the Faradaic reaction occurs at the edge of the AgCl deposit and Ag surface,^1^ meaning that some exposed Ag surface is necessary for the electrode to manifest low resistance, these holes are considered to actually form channels leading to the Ag wire surface like microchannels so that the electrode fabricated under a constant current of 4 mA for 3600 s manifested lower resistance (Ω < 10) after some initial impedance measurements than that of the as-prepared electrode.

A comparison of Figure 9 with Figure 3f together with the results (Figure 2h, Figure 4a) from the electrode with the theoretical AgCl thickness of 1.9 μm further suggests that some exposed Ag surface is necessary for the electrode to manifest Ω < 10 to facilitate the Faradaic reaction. When simply looking at the data in Figure 7a, all the electrodes with a theoretical AgCl thickness of 1.9 μm can be the best choice for the LFEFD sensor in terms of the resistance value. However, as described above, the resistance of the electrodes exceeded 10 Ω just after less than 10 times repeats of the impedance measurements, suggesting that the electrodes may not be good for long-term use in actual applications. Although electrodes with thicker AgCl layers manifested resistance value Ω > 10 (a few tens of Ω) during some initial measurements, they kept Ω < 10 over the subsequent many more measurements than the number of measurements where the electrodes with a theoretical AgCl thickness of 1.9 μm showed Ω < 10 in Figure 7b. The number of measurements for the electrodes fabricated under the constant currents of 4 mA keeping Ω < 10 was proportional to the theoretical AgCl thickness as seen in Figure 7d, which may indicate that the dissolved AgCl amount upon each impedance measurement was very similar among the three electrodes. The average resistance values of all these three electrodes when they manifested Ω < 10 are displayed in Figure 7e. The resistance values among them are very similar when they manifested Ω < 10. Then, these results from Figure 7d,e suggest that using the electrode with a theoretical AgCl thickness of 7.62 μm just having Ω < 10 after some initial impedance measurements will be the best choice among the tested electrodes for the application to the detection of low-frequency electric field signals in seawater in terms of both sensitivity (Ω < 10 for bare electrode) and service lifetime.

## Conclusions

In this study, we have investigated the sequential AgCl formation mechanism on the Ag wire in a dimension of 2 mm × 11 cm, an actual electrode size designed for the application to the marine LFEFD Ag/AgCl sensor and proposed a desired AgCl structure for the sensor electrode based on the results of resistance at 1 Hz—from the impedance measurements—deriving from the specific AgCl structures on the electrode surfaces.

The AgCl deposits are found to form via nucleation, as reported by many previous studies. The unique point of our study is that we reveal the sequential AgCl formation process from the beginning to full AgCl coverage in an electrode size for actual applications in much detailed descriptions that are lacking in conventional studies. The process is summarized as follows. At the beginning of anodization under a mild condition of the constant current of 1 mA (i.e., 0.2 mA/cm^2^) in 3.3 M KCl solution, the induction period in which potential little increases with time develops in the anodization graph. During this period when Ag is dissolved, grains on the Ag wire surface grow and AgCl deposits or particles in sizes of about several micrometers to 10 μm with crystal planes also form along scratch lines—preferred heterogeneous sites—on the wire surface, but in a partial scale. Then, the assumed thin AgCl deposits begin to form and cover a large portion of the wire surface, as judged from the analyses by the SEM/EDX and XRD measurements together with the abrupt potential rise in the anodization graph, indicating an increase in resistance. With continued anodization, they grow to become AgCl deposits in sizes of about several micrometers to 10 μm with no manifestations of clear crystal planes and are connected together to make the network structure with distinct boundaries in it, covering the wire surface. This represents the main developing mode of the AgCl deposits. Other AgCl deposits form on the surface of these AgCl deposits, subsequently leading to the formation of multiple AgCl layers.

The observed increase of the amount of the AgCl deposits with anodization time and the applied current magnitude via SEM images is well reflected in XRD data. The analyses of induction time with respect to the applied current magnitude and of the Cl^–^ concentration nearby the wire surface with the anodization time by IC suggest that the AgCl deposits form via nucleation with a relatively high nucleation barrier (1586 erg/cm^2^ if the spherical nucleus shape is assumed) and that the subsequent growth is solely controlled by the release rate of Ag^+^ from the wire, thus by the applied current magnitude.

The resistance (Avg. about 6 Ω) of the Ag/AgCl electrodes at 1 Hz—an important parameter for the electrochemical performance of the LFEFD sensor electrodes—is not affected by the current magnitudes applied to the fabrication of the electrodes for the same theoretical AgCl thickness of 1.9 μm (by Faraday’s law) but increases with the theoretical AgCl thickness. Although the electrode with the thin AgCl layer manifests low resistance due to the facilitated Faradic reaction, leading to an excellent sensitivity, it will not be suitable for the long-term service lifetime. It turns out that the electrode with a thick AgCl layer followed by a process to reduce some AgCl layer that makes many holes in the AgCl structure like microchannels will work effectively in terms of both sensitivity and service lifetime.

## Experimental Section

### Materials

Ag wires (diameter: 2 mm) with purity over 99.99% and KCl (99.5% (GR)) were purchased from Inexus and JUNSEI, respectively. The bottom of the Ag wire cut to the desired length (11 cm) was polished by silicon carbide abrasive paper (60 Cw), and then the wire was sonicated for 20 min followed by washing with distilled water (DW) and dried for anodization.

### Fabrication of Ag/AgCl Electrodes

The two-electrode system was used. Ag wire and platinum (Pt) foil, apart 4 cm from each other, were connected to the working electrode probe and to the counter electrode probe, respectively. Anodization rendering deposition of the AgCl layer on Ag wire (diameter: 2 mm) was carried out in 3.3 M KCl solution (250 mL) at room temperature by the constant current method using an electrochemical workstation (PGSTAT302N, Metrohm Autolab B.V.). Various constant currents (1, 2, 4, and 8 mA) were applied to the length of 8 cm of the Ag wire with a whole length of 11 cm, which translated to respective current densities of (0.2, 0.4, 0.8, and 1.6 mA/cm^2^).

### SEM Imaging

Imaging was performed by using a field emission SEM (JSM-7600F, JEOL, Japan). An Ag wire cut at about 1 cm from its one end was placed onto the carbon tapes attached to the SEM holder, and the surface structure on the circumferences of the wire was observed.

### EDX Analysis with SEM

X-Max EDS (Oxford Instruments, UK) was used to obtain the atomic percentage of elements on the structures of the Ag wire surface.

### XRD Analysis

The AgCl deposits formed on the surfaces of the anodized wires were analyzed by XRD (D8 Advance, Bruker Inc., Germany) using Cu-Kα radiation (λ = 1.5406 Å).

### IC Analysis of Chloride Ions from Electrolyte Solution during Anodization

An ion chromatographer (Dionex DX-120 and Dionex Aquion, Thermo Fisher) equipped with a Dionex IonPac AS 22 (4 × 150 mm) together with a conductivity detector was employed to perform the anion chromatography. A solution of Na_2_CO_3_ (4.5 mM)/NaHCO_3_ (1.4 mM) was used as an eluent for anion chromatography. To examine Cl^–^ concentration in the solution near the Ag wire at reaching a specific time during the anodization, an aliquot of 25 μL was taken from the solution near the wire at reaching the time. Then, this aliquot was diluted into DW so as to be a total of 50 mL. Then 1.2 mL was taken out of this solution and mixed with DW of 8.8 mL to be a total of 10 mL. Then 500 μL was taken from this prepared solution and Cl^–^ concentration in it was measured. Aliquots for specific anodization times were prepared from two independent measurements.

### Resistance Measurements of Ag/AgCl Electrodes

The three-electrode testing system was used for the measurements. The working, reference, and counter electrodes of the testing system were the fabricated Ag/AgCl, commercial Ag/AgCl reference (MF-2052; Qrins), and Pt foil electrodes, respectively. Each electrode was apart 4 cm from one another in a beaker containing 3.5% NaCl 250 mL solution; the fabricated Ag/AgCl electrode was immersed in 3.5% NaCl solution for 24 h before the impedance measurements. The measurements were conducted by applying the sine wave excitation signal of 10 mV in the frequency range of 0.01–1000 Hz, and resistance values of the electrodes at 1 Hz were compared because the value of low-frequency impedance is an important parameter for the application of the electrodes to the detection of the weak low-frequency electric field signal in seawater. Average resistance values were obtained from five tested electrode samples for each fabrication condition.

## Supporting Information Available

Calculation of the theoretical AgCl thickness on the Ag wire surface by Faraday’s first law, SEM images of the surface of the Ag wire before being applied to anodization, graphs of the anodization of the Ag wire at a constant current of 1 mA for 1 h using the two-electrode system, SEM images of the AgCl deposits formed along scratch lines on the Ag wire surface during anodization of 1070 s under a constant current of 1 mA, and graph of the anodization of the Ag wire by the three-electrode setup under a constant current condition of 1 mA for 1 h (PDF)
