# Supplementary material for: Investigation of the AgCl Formation Mechanism on the Ag Wire Surface for the Fabrication of a Marine Low-Frequency-Electric-Field-Detection Ag/AgCl Sensor Electrode
Source: ACS Omega. 2022 Jun 24;7(29):25110–21. doi: 10.1021/acsomega.2c01481 (PMC9330149; doi:10.1021/acsomega.2c01481)
Supplement: Supplementary file 1 — ao2c01481_si_001.pdf [file ao2c01481_si_001.pdf]

Investigation of the AgCl Formation Mechanism on the Ag Wire Surface for the Fabrication of a Marine Low-Frequency-Electric-Field-Detection Ag/AgCl Sensor Electrode

Kang Rae Cho,[1#*] Minhye Kim,[1#] Bupmo Kim,[2] Gahye Shin,[1] Sangkyu Lee,[3] and Wooyul Kim[1*]

[1]Department of Energy Engineering / KENTECH Institute for Environmental and Climate Technology, Korea Institute of Energy Technology (KENTECH), Naju 58330, Republic of Korea
[2]Department of Chemical Engineering & Division of Environmental Science and Engineering, Pohang University of Science and Technology, Pohang 37673, Gyeongbuk, Republic of Korea
[3]Maritime Technology Research Institute 1st Directorate Agency for Defense Development, Jinhae-gu, Changwon-si 51698, Republic of Korea

[#]These authors contributed equally
*To whom correspondence should be addressed: K.R.C.(kangraecho@kentech.ac.kr); W.K.(wkim@kentech.ac.kr)

Discussion S1. Calculation of theoretical AgCl thickness by Faraday's first law

The thickness of the AgCl layer deposited on the Ag surface is theoretically approximated by equation S1, which represents Faraday's first law:[3]

$$X = \frac{\left(\frac{I}{A}\right)Mt}{Fd} \qquad \text{(S1)}$$

where X is the thickness of deposited AgCl layer (cm), I is applied current (C/sec), A is the electrode (i.e., wire) surface area where AgCl is deposited (cm^2), M is the molecular weight of AgCl (143.5g/mol), t is the duration of anodization (sec), F is Faraday's constant (F = 96500 C/mol/equiv) and d is the density of the AgCl layer (5.56 g/cm^3).

Since AgCl is deposited onto the circumference and bottom area of the Ag wire with a length of 8 cm and diameter of 2 mm, A is approximated to be 5.0554 cm^2. With 1 mA, the obtained value of A, and the values of other components in equation S1, X = 1.9 μm for t = 3600 sec, X = 3.8 μm for t = 7200 sec, X = 5.7 μm for t = 10800 sec and X = 7.6 μm for t = 14400 sec. With 4 mA, X = 7.6 μm for t = 3600 sec. Thus, theoretically by equation S1, the condition of 4 mA for 1 hour has the same thickness or amount of deposited AgCl as the condition of 1 mA for 4 hours.

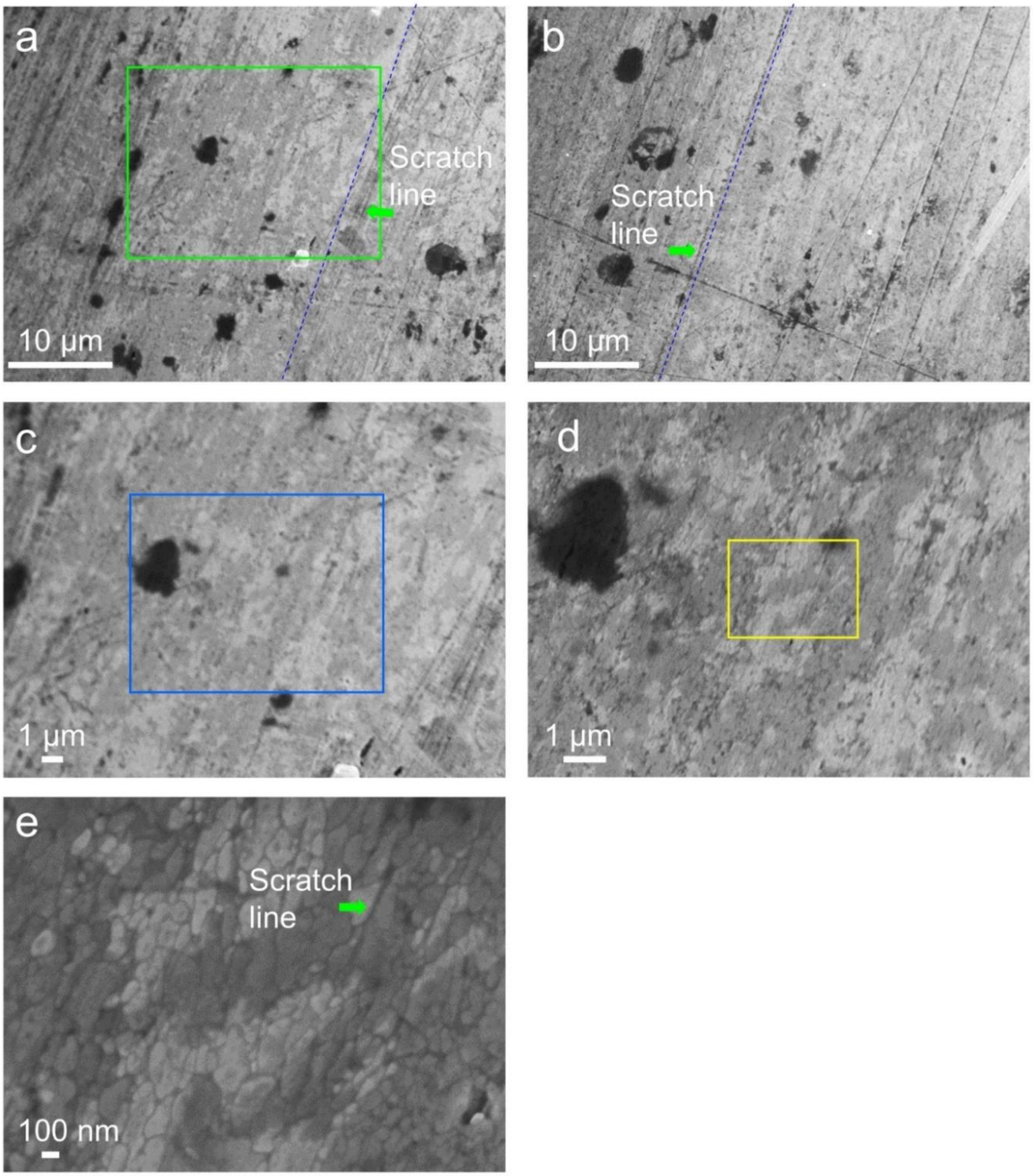

Figure S1. SEM images of the surfaces of the Ag wire before being applied to anodization. (a, b) Images showing scratch lines along which AgCl deposits with crystal planes formed during the initial stage of anodization. Blue dotted lines schematically imitate the scratch lines next to them. See Figure S3 for the image of the AgCl deposits with crystal planes formed along with the scratch line directions. (c) Magnified view of the area within a green box in (a). (d) Magnified view of the area within a blue box in (c). (e) Magnified view of the area within a yellow box in (d).

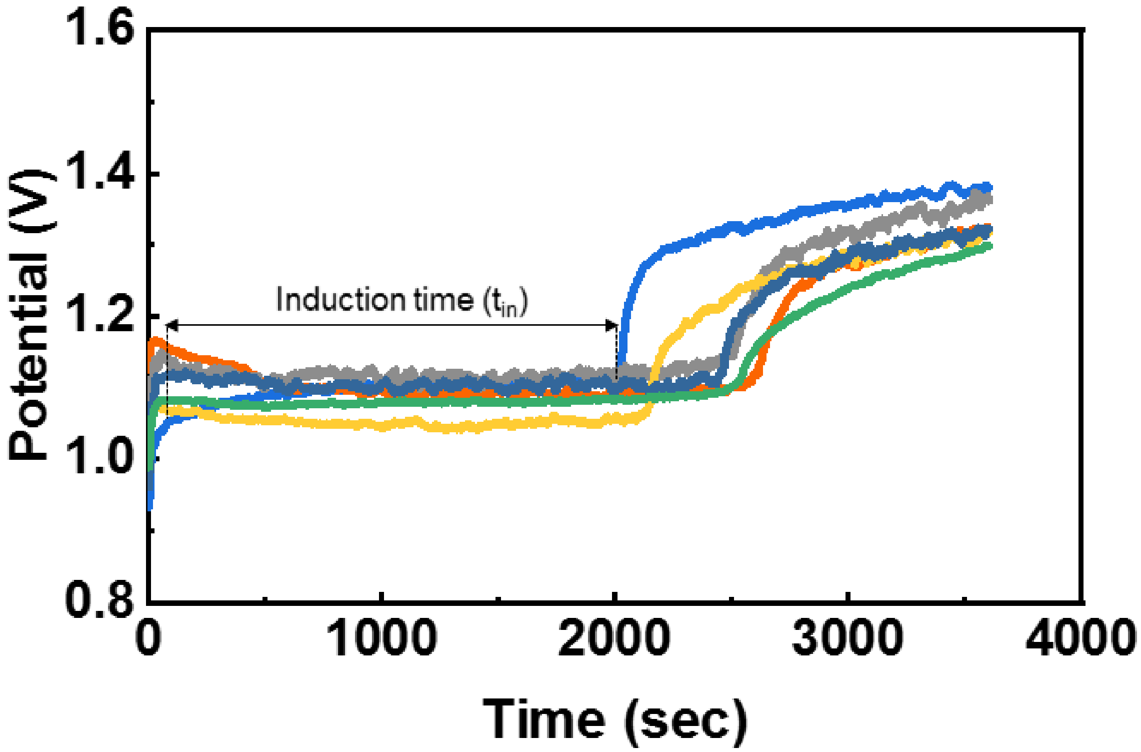

Figure S2. Graphs of the anodization of the Ag wire at a constant current of 1 mA for 1 hour. The results from the independent experiments show that there is some deviation in the length of induction time for each experiment.

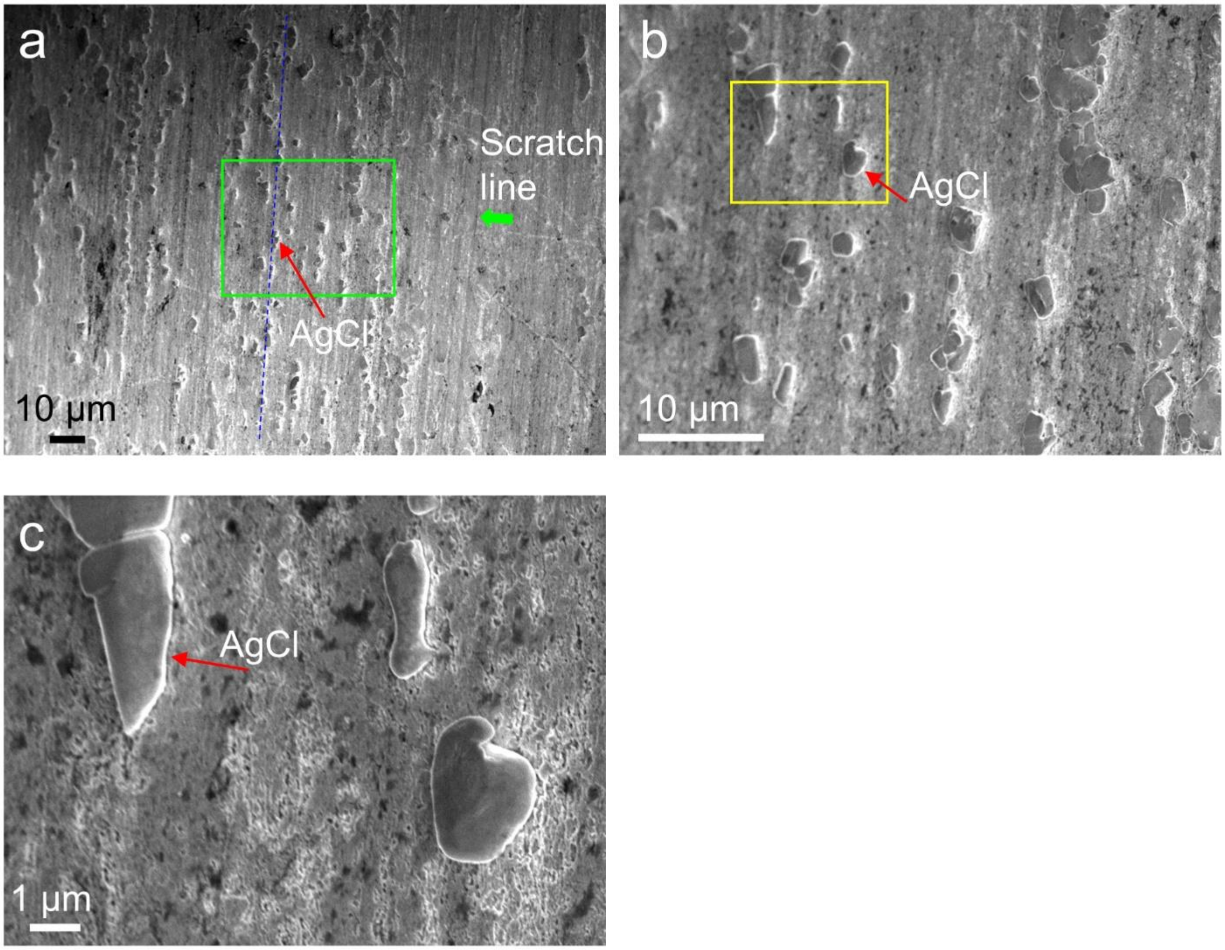

Figure S3. SEM images of the AgCl deposits formed along scratch lines on the Ag wire surface during anodization of 1070 sec under the constant current of 1 mA. (a) Formation of the AgCl deposits with crystal planes along scratch line directions. For example, see the deposits aligned along with the dotted blue line. (b) Magnified view of the area within a green rectangle in (a). (C) Magnified view of the area within a yellow rectangle in (b).

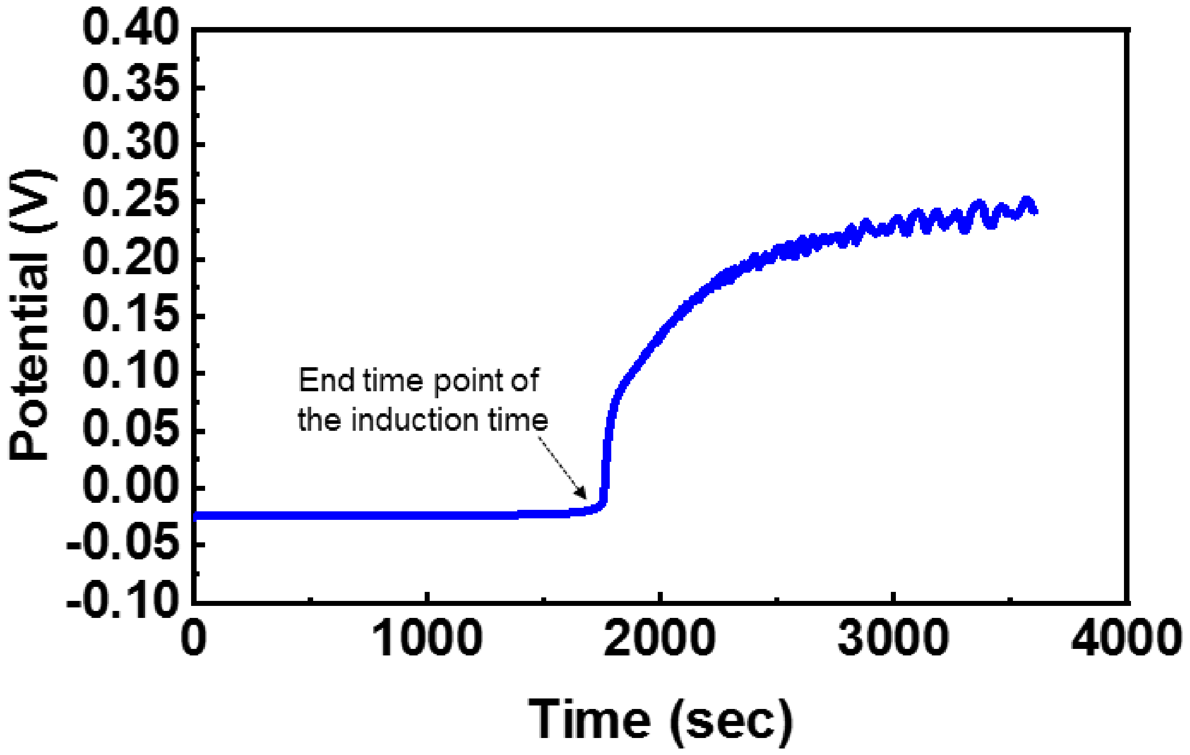

Figure S4. Graph of the anodization of the Ag wire by the three electrodes setup that uses the Ag wire as the anode electrode, standard Ag/AgCl as the reference electrode and platinum foil as the counter electrode under the constant current condition of 1 mA.

Acquisition of E_{RHE} (V vs. RHE) against the reversible hydrogen electrode at the end time point of the induction time: E_{RHE} (V vs. RHE) is obtained by inserting $E_{Ag/AgCl}$ (-0.016 V vs. Ag/AgCl) against the Ag/AgCl reference electrode at the end of induction time in the above graph and pH 9.5 into the following equation:[S1]

$$E_{RHE} = E_{Ag/AgCl} + 0.059pH + E^{0}_{Ag/AgCl}$$

where $E^{0}_{Ag/AgCl}$ is the standard potential of Ag/AgCl (KCl 3 M) at 25 °C (*i.e.* 0.21 V).
The obtained E_{RHE} is estimated to be 0.75 V.

Supporting Reference

S1. Hernández, S.; Hidalgo, D.; Sacco, A.; Chiodoni, A.; Lamberti, A.; Cauda, V.; Tresso, E.; Saracco, G. Comparison of Photocatalytic and Transport properties of TiO_2 and ZnO Nanostructures for Solar-Driven Water Splitting. *Phys. Chem. Chem. Phys.*, **2015**, *17*, 7775–7786.
